# Pathogen-Reactive T Helper Cell Analysis in the Pig

**DOI:** 10.3389/fimmu.2017.00565

**Published:** 2017-05-17

**Authors:** Friederike Ebner, Patrycja Schwiertz, Svenja Steinfelder, Robert Pieper, Jürgen Zentek, Nicole Schütze, Christoph G. Baums, Gottfried Alber, Peter Geldhof, Susanne Hartmann

**Affiliations:** ^1^Department of Veterinary Medicine, Institute of Immunology, Freie Universität Berlin, Berlin, Germany; ^2^Department of Veterinary Medicine, Institute of Animal Nutrition, Freie Universität Berlin, Berlin, Germany; ^3^Faculty of Veterinary Medicine, Institute of Immunology, Centre for Infectious Diseases, University of Leipzig, Leipzig, Germany; ^4^Faculty of Veterinary Medicine, Institute for Bacteriology and Mycology, Centre for Infectious Diseases, University of Leipzig, Leipzig, Germany; ^5^Laboratory of Parasitology, Faculty of Veterinary Medicine, Ghent University, Merelbeke, Belgium

**Keywords:** antigen-specific, pig, porcine CD4 T cell, CD154, CD40 ligand, *Ascaris suum*, *Candida albicans*, *Streptococcus suis*

## Abstract

There is growing interest in studying host–pathogen interactions in human-relevant large animal models such as the pig. Despite the progress in developing immunological reagents for porcine T cell research, there is an urgent need to directly assess pathogen-specific T cells—an extremely rare population of cells, but of upmost importance in orchestrating the host immune response to a given pathogen. Here, we established that the activation marker CD154 (CD40L), known from human and mouse studies, identifies also porcine antigen-reactive CD4^+^ T lymphocytes. CD154 expression was upregulated early after antigen encounter and CD4^+^CD154^+^ antigen-reactive T cells coexpressed cytokines. Antigen-induced expansion and autologous restimulation enabled a time- and dose-resolved analysis of CD154 regulation and a significantly increased resolution in phenotypic profiling of antigen-responsive cells. CD154 expression identified T cells responding to staphylococcal Enterotoxin B superantigen stimulation as well as T cells responding to the fungus *Candida albicans* and T cells specific for a highly prevalent intestinal parasite, the nematode *Ascaris suum* during acute and trickle infection. Antigen-reactive T cells were further detected after immunization of pigs with a single recombinant bacterial antigen of *Streptococcus suis* only. Thus, our study offers new ways to study antigen-specific T lymphocytes in the pig and their contribution to host–pathogen interactions.

## Introduction

Pigs are not men, but closer related to men than any other rodent model in many aspects of anatomy, genetics, immunology, and physiology (1–4). Porcine immune-related organs display some organizational particularities compared to humans, e.g., inverted lymph node structures, a continuous ileal Peyer’s patch and a six-layered epitheliochorial placenta (5, 6). However, in terms of blood immune cell composition, innate immune cell function and pattern-recognition receptors, functional T cell subsets, and cytokines numerous publications recently reported striking similarities to its human counterparts, altogether concluding that the pig is a highly valuable model to study human infectious disease (3, 7–9).

Researchers being convinced of the benefits of the pig as a large animal model are still confronted by additional requirements and higher costs for animal facilities, breeding, or substance application, but more importantly by the lack of crucial immunological tools or suitable reagents to comprehensively study the host–pathogen relationship in pigs. However, especially in the field of T cell biology, there has been remarkable progress in developing pig-specific and cross-reactive antibodies for multicolor flow cytometry (8, 10–14), in diagnostic bead-based multiplex assays (15) as well as in annotating the porcine immune system (16). These efforts helped to expand the opportunities for studying adaptive immunity in this human-relevant large animal model.

The quality of an immune reaction to a given pathogen largely depends on a relatively small population of antigen-specific T lymphocytes (17, 18). Frequency, phenotype, and functional capacities of those cells have been proven to orchestrate inflammation, regulation, and protection in response to pathogen encounter. Understanding the role of antigen-specific T cells in the host immune response is therefore crucial to evaluate host–pathogen interactions and provides a powerful tool for diagnostics, therapy, or for developing effective T cell-dependent vaccines (17, 19, 20). Due to the very low abundance of antigen-specific T cells in the absence of an infection [1 in 10^−6^ within naïve and 1–100 in 10^−5^ within the memory compartment of conventional T cells (21, 22)] studying their functional potential remains difficult. For human studies, several ways exist to identify antigen-specific T lymphocytes directly or indirectly for further characterization. These include the use of MHC–peptide multimers and antibody-based methods such as ELISPOT, intracellular cytokine staining (ICS), or antigen-specific cultivation followed by the analysis of defined activation markers (23, 24). One activation marker is CD154 (CD40L), a member of the TNF superfamily and upregulated following short-term (5–7 h) *ex vivo* stimulation (25–27). CD154 is transiently expressed by conventional CD4 T cells after T cell receptor stimulation (TCR), but immediately internalized after binding to CD40 expressed on antigen-presenting cells (APCs) (28–30). MHC restriction of CD154 was previously shown by blocking human HLA-DR during antigen stimulation or in TCR-transgenic mice (27, 31). The analysis of antigen-reactive T cells in humans based on CD154 expression is robust, highly sensitive, and combined with enrichment technologies enables the characterization of T cell subset composition in high resolution and with low intra-assay variability (22, 32).

In swine influenza or circovirus studies, researchers already applied *ex vivo* restimulation, but focused only on the cytokine responses to a given antigen by ICS (9, 33, 34). Pigs are natural hosts for several important zoonotic pathogens infecting humans alike [e.g., *Streptococcus suis, Ascaris suum*, and *Toxoplasma gondii* (35–37)]. In addition, pigs exhibit very similar pathologies (3, 8) and are of importance for vaccine development for pigs and human likewise. Hence, tools for an extended multiparameter analysis of rare pathogen-specific T cells are of great importance.

Despite being now routinely used in mouse and human systems to address rare antigen-specific T cell populations, the potential of using CD154 in pigs as a reliable marker of antigen-specific T lymphocytes has, to the best of our knowledge, not yet been investigated. We therefore evaluated whether CD154 expression identifies antigen-reactive CD4^+^ T cells in pigs upon staphylococcal enterotoxin B (SEB) stimulation and in response to lysates of *Candida albicans* (*C. albicans*), a commensal fungus of pigs and men (38–41). Our data show that also in the absence of an acute infection, CD154 expression on pig CD4^+^ T cells occurs within 4 h after antigen activation, that CD4^+^CD154^+^ cells derive primarily from the activated/memory but also the naïve T cell compartment and that they coexpress cytokines. We refined our analysis by combination of antigen-specific expansion and autologous restimulation with the given antigen to examine time- and dose-resolved regulation of CD154 expression and to increase the resolution in phenotypic profiling.

Furthermore, in the context of an intestinal parasitic roundworm infection with *Ascaris* sp., which is highly prevalent in pigs and men, we could prove organ-specific accumulation of antigen-activated T cells identified by CD154 in the tissues being affected by larval migration. Our data further reveal that using CD154 marker expression identifies immunization-responsive cells specific for a single recombinant protein from *S. suis* and can therefore also be applied to validate the induction of a T helper cell response toward single proteins, such as subunit vaccines in swine. Thus, we successfully detected and functionally analyzed CD154-expressing CD4^+^ T lymphocytes specific for SEB, *C. albicans, A. suum*, and *S. suis* in steady state and after infection and immunization.

## Materials and Methods

### Animals, Sampling, and Necropsy

For analyzing *C. albicans*- or superantigen (SEB)-reactive CD4^+^ T cells, blood was sampled from healthy German Landrace pigs (3–12 months) either by taking blood from the external jugular vein or by heart puncture after sedation with ketamine hydrochloride and azaparone (20 mg/kg BW; Ursotamin; Serumwerk Bernburg AG and 2 mg/kg BW; Stresnil; Janssen-Cilag GmbH).

For analyzing *A. suum*-reactive CD4^+^ T cells during acute, primary parasite infection, German landrace piglets were aged 8–10 weeks and orally inoculated with a dose of 12–15,000 *A. suum* eggs/pig. Parts of spleen and lung were sampled from infected piglets after sedation with ketamine hydrochloride and azaparone (20 mg/kg BW; Ursotamin; Serumwerk Bernburg AG and 2 mg/kg BW; Stresnil; Janssen-Cilag GmbH) and euthanizing the animals by intracardial injection with 10 mg/kg BW of tetracaine hydrochloride, mebezonium iodide, and embutramide (T61, Intervet, Germany).

For analyzing *A. suum*-reactive CD4^+^ T cells during trickle infection, commercial Rattlerow Seghers hybrid piglets were aged for 4 weeks. Animals were orally inoculated with 80 *Ascaris* eggs per day for seven consecutive weeks. At the end of this study, all piglets were first sedated with Stresnil (Janssen-Cilag GmbH) and subsequently euthanized by electric stunning followed by exsanguination, and parts of the spleen and lung were sampled.

For analyzing *S. suis-*reactive CD4^+^ T cells after Ide*_Ssuis_* immunization, German Landrace piglets at the age of 5–12 weeks were intramuscularly injected as described (42) with 0.4 mg recombinant His-tagged Ide*_Ssuis_* (rIde*_Ssuis_*, prime) and boosted with 0.25 mg rIde*_Ssuis_* 2 weeks later, supplemented with 20% (vol/vol) Emulsigen (MVP Technologies, Omaha, NE, USA) as adjuvant. Placebo control animals were exclusively injected with PBS, supplemented with 20% (vol/vol) Emulsigen. Fourteen days post-booster immunization, heparinized blood samples were taken from the *Vena cava cranialis*.

### Generation of Parasitic Antigens and Infective Material

Infective *A. suum* eggs were produced as previously described (43). In brief, *A. suum* eggs were obtained by culturing female adult worms from the slaughter house overnight in worm culture medium [BSS supplemented with 1% Glucose (AppliChem), 200 U/ml Penicillin and 200 µg/ml Streptomycin (PAN-Biotech), Gentamycin (50 µg/ml, PAN-Biotech), and Amphotericin B (0.25 µg/ml, PAN-Biotech)]. Released eggs were collected, washed several times in water, and placed in 0.1% formalin-containing distilled water for embryonation (4 weeks). Embryonation rates were checked weekly and by reaching 95% fertilized eggs used for infection. For generation of worm antigens (Asc Lys) and worm excretory–secretory products (Asc ES), *A. suum* L3 larvae were recovered from the lungs of infected animals using a modified Baermann funnel. Therefore, lungs were cut into 1–2 cm chunks and placed in 37°C pre-warmed saline (0.9%) onto a gaze-lined funnel. Vital worms were isolated from the flow through, washed extensively in antibiotic containing worm culture media, and were either snap-frozen for production of L3 worm lysate (Asc Lys) or cultured in worm culture media for 1–2 weeks to collect excretory–secretory products containing worm culture supernatants. Worm culture supernatants were further concentrated using centrifugal protein concentrators with a 5-kDa MWCO (Vivaspin, Sartorius) to obtain the final, concentrated Asc ES. LPS contaminations of Asc Lys and Asc ES were analyzed by LAL test using Endosafe^®^-PTS™ and cartridges (Charles River Laboratories International, Inc.). Calculated as final concentration when applied *in vitro* LPS values ranged between 0.812 and 1.624 ng/ml for Asc Lys and 0.0261 and 0.0522 ng/ml for Asc ES.

### Leukocyte Isolation

Mononuclear cells from porcine peripheral blood were isolated by density centrifugation of diluted (1:2 in 0.9% NaCl) blood using Pancoll solution (density 1.077 g/ml, PAN-Biotech). Splenic cells were isolated by passing spleen tissue through a 70-µm mesh, followed by erythrocyte lysis with ACK buffer containing 150 mM NH_4_Cl, 10 mM KHCO_3_, and 0.1 mM Na_2_EDTA. Gradient isolation of lung lymphocytes was performed after enzymatic tissue digestion. In brief, lung tissue was rinsed with warm HBSS (PAN-Biotech) using a 20-gauge needle penetrating several areas to bleed out the organ sample. Pre-digestion was performed by introducing collagenase VIII (Sigma-Aldrich) and collagenase D (Sigma-Aldrich) in warm HBSS (PAN-Biotech) supplemented with 2% FCS (PAN-Biotech) and 0.6% BSA (AppliChem) using again a 20-gauge needle. The organ was cut into little pieces, transferred to Liberase TM/DH (1:2, Sigma-Aldrich) and DNase I (0.15 mg/ml, Sigma-Aldrich) containing HBSS, and digested at 37°C for 30 min. Ice-cold HBSS was used to stop digestion, and the suspension was filtered using a 70-µm cell strainer to remove cell clumps and undissociated tissue. After washing, the cell pellet was resuspended in 40% Percoll solution (GE healthcare™) and layered onto 70% Percoll. After gradient isolation at 400 × *g* for 20 min lymphocytes were obtained at the interface. Porcine CD14^+^ monocytes were purified from PBMC or splenocytes using magnetic bead separation (CD14 MicroBeads, Miltenyi Biotec).

### Antigen Activation *In Vitro*

For detection of SEB or *C. albicans*-reactive Th cells from healthy pigs, isolated PBMCs were rested overnight in complete IMDM (PAN-Biotech) supplemented with 10% FCS (PAN-Biotech), 100 U/ml Penicillin, and 100 µg/ml Streptomycin (PAN-Biotech) and stimulated on the following day with superantigen (SEB, 1 µg/ml, Sigma-Aldrich) or PMA (20 ng/ml, Sigma-Aldrich) plus ionomycin, (1 µg/ml, Sigma-Aldrich) or *C. albicans* lysate (40 µg/ml, GREER^®^) for 6 h and in presence of BrefeldinA (3 µg/ml, eBioscience) during the last 4 h of restimulation. *C. albicans* lysate was produced by dissolving statically grown cellular antigen, defatted, powdered, and dried by GREER^®^ in PBS. For detection of *A. suum*-reactive Th cells, splenocytes and lung parenchymal cells were rested overnight in complete IMDM (PAN-Biotech) supplemented with 10% FCS, 100 U/ml Penicillin, and 100 µg/ml Streptomycin and stimulated on the following day with 20 µg/ml of *Ascaris* L3 worm lysate (Asc Lys) or L3 excretory–secretory products (Asc ES) for 6 h and in presence of BrefeldinA (3 µg/ml, eBioscience) during the last 4 h of restimulation.

### Cellular Immunophenotyping

Cells were stained for flow cytometry analyses (BD FACS Canto II, BD FACS Diva software, FlowJo v9 software by Tree Star) with the following antibodies specific for pig species: anti-TCR1δ-unlab (clone PGBL22A, IgG1, Kingfisher Biotech), anti-CD4a-Pe-Cy7 or -AlexaFluor^®^ 647 or -PE (clone 4-12-4, IgG2b, BD Biosciences), anti-CD3ε-PerCP-Cy5.5 or -PE-Cy7 (clone BB23-8E6-8C8, IgG2a, BD Biosciences), anti-CD8α-FITC or -AlexaFluor^®^ 647 or -PE (clone 76-2-11, IgG2a, BD Biosciences), and IFN-γ-PE or -PerCP-Cy5.5 (clone P2G10, IgG1, BD Biosciences).

The following cross-reactive primary or secondary antibodies were used: anti-human CD14-Viogreen (clone Tük4, IgG2a, Miltenyi Biotec), anti-human CD154-Vioblue or, -PE (clone 5C8, IgG2a, Miltenyi Biotec), anti-human TNF-α-Pacific Blue or -Brillant Violet 605 (clone Mab11, IgG1, BioLegend) anti-human IL-17A-AlexaFluor^®^ (clone CZ8-23G1, IgG1, Miltenyi Biotec), and anti-mouse IgG1-FITC (clone M1-14D12, IgG, eBioscience). Fixable viability dyes were used in eFluor^®^ 780 or 506 and CFSE as proliferation dye (all from eBioscience). Intracellular antigens were stained after fixation and permeabilization of cells (Transcription Factor Staining Buffer Set, eBioscience or Cytofix/Cytoperm™, BD).

### Expansion and Autologous Restimulation

CFSE-labeled porcine PBMC from healthy animals were expanded in complete IMDM supplemented with 10% FCS (PAN-Biotech), 100 U/ml Penicillin, and 100 µg/ml Streptomycin (PAN-Biotech) in the presence of either *C. albicans* lysate (20 µg/ml, GREER) or SEB (1 µg/ml, Sigma-Aldrich) for 7 days followed by two resting days. In parallel, autologous CD14^+^ monocytes were seeded in complete IMDM at an initial density of 2.5 × 10^5^ cells/well in 96-well plates and differentiated into monocyte-derived dendritic cells (MoDC) in the presence of recombinant pig GM-CSF (20 ng/ml, R&D) and interleukin (IL)-4 (10 ng/ml, R&D) for 7 days. MoDC were primed with *C. albicans* antigen (if not indicated elsewhere: 40 µg/ml) or left unprimed for 1 day and matured with LPS (100 ng/ml), recombinant pig TNF-α (25 ng/ml) and pig recombinant IL-1β (10 ng/ml) for another day. For *C. albicans*-specific restimulation, expanded pig PBMC were harvested, washed, and 400,000 cells/200 μl/well were added to primed and matured MoDC for 6 h. Brefeldin A (3 µg/ml, eBioscience) was added for the last 4 h of autologous restimulation. Notably, for analyzing superantigen-reactive T cells, SEB-expanded cells were restimulated with unprimed, matured MoDC in the presence of SEB antigen (1 µg/ml).

### Detection of Ide_*Ssuis*_-Reactive Th Cells

For *ex vivo* detection of Ide*_Ssuis_*-reactive Th cells, PBMCs were separated from heparinized blood by density gradient centrifugation on Biocoll (Merck-Biochrome). Samples were cryo-conserved in FCS, 10% DMSO at −80°C, or liquid nitrogen. Thawed PBMCs were cultivated in RPMI (Merck-Biochrome), supplemented with 10% FCS, 100 U/ml Penicillin, and 100 µg/ml Streptomycin. Following a resting time (2–4 h), PBMCs were stimulated for 18 h with 5 µg/ml rIde*_Ssuis_* or with control-antigen (ctr-ag) Sfb I (fibronectin-binding protein of *Streptococcus pyogenes*), and in presence of Brefeldin A (2 µg/ml, Enzo Life Sciences) for the last 4 h. Recombinant Ide*_Ssuis_* was expressed in *E. coli* and purified as described previously (42). As control antigen, the His-tagged C-terminal part of the *S. pyogenes* SfbI protein expressed in *E. coli* M15 pSTH 12 was used (44). LPS contaminations of rIde_*Ssuis*_ and rSfbI were analyzed by LAL test using Endosafe^®^-PTS™ and cartridges (Charles River Laboratories International, Inc.). Final LPS concentration applied *in vitro* ranged between 0.118 and 0.236 ng/ml for rSfbI and 0.95 and 1.9 ng/ml for rIde_*Ssuis*_. 3–5 × 10e5 CD3^+^CD4^+^ cells/sample were measured with BD LSRFortessa, and data analysis was performed using FlowJo V10.1 (Tree Star Inc., OR, USA) and GraphPad PRISM^®^V5.01 software (La Jolla, CA, USA).

### Analysis of Ide_*Ssuis*_-Specific IgG

Serum IgG antibodies specific for rIde_*Ssuis*_ were determined in an ELISA as described previously (42).

## Results

### Porcine Antigen-Reactive CD4^+^ T Cells Express CD154

**Figure 1:**
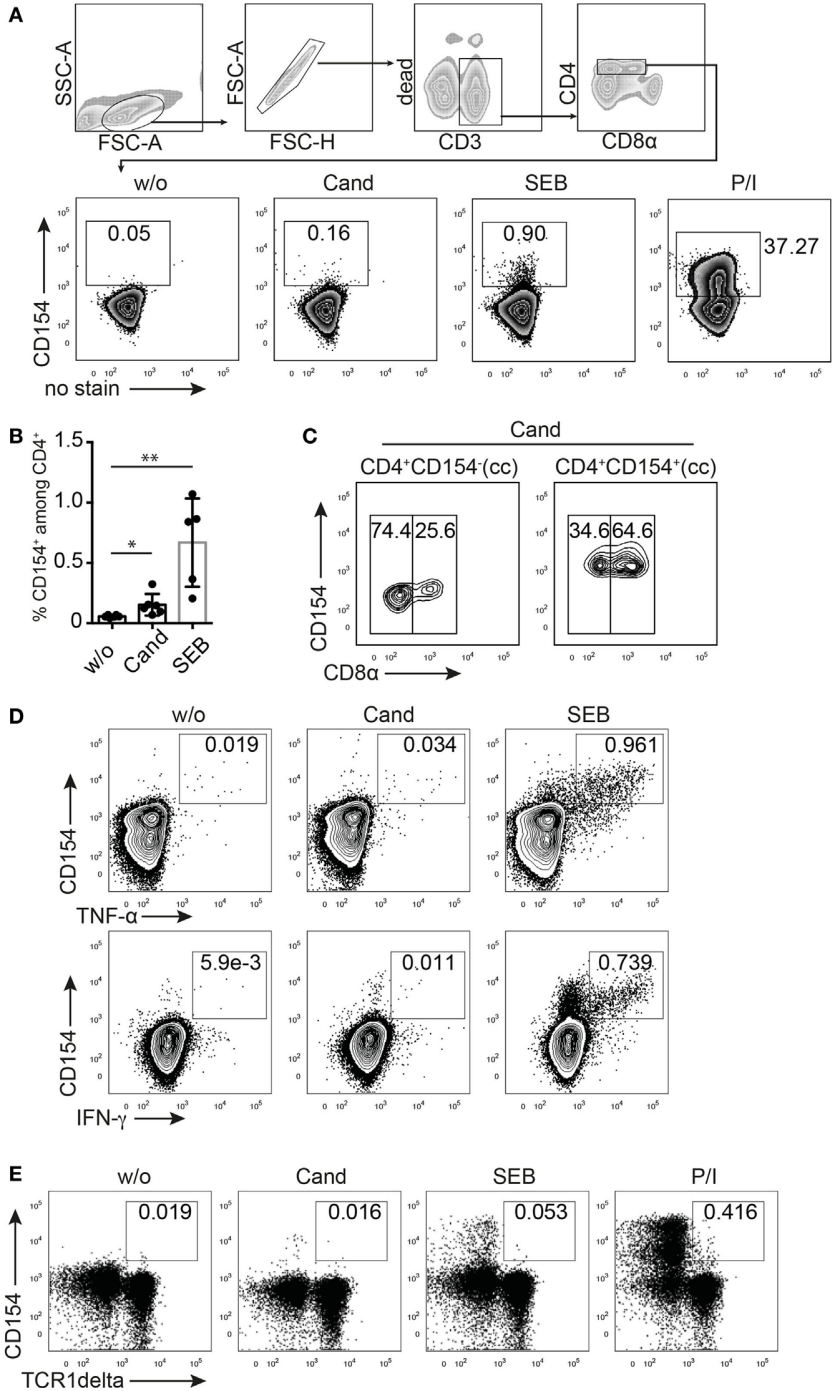
**CD154 identifies porcine, antigen-reactive CD4^+^ T cells**. T cell receptor stimulation (TCR)-independent and TCR-dependent activation induced CD154 expression in pig CD4^+^ T cells detected by flow cytometry and intracellular staining. **(A)** Gating strategy and representative zebra plots showing the CD154 signal from *ex vivo* PBMC gated on CD4^+^ T cells that were either unstimulated (w/o) or stimulated with *Candida albicans* lysate (Cand, 40 µg/ml), staphylococcal Enterotoxin B (SEB, 1 µg/ml), or PMA/ionomycin (P/I) for 6 h. Panel **(B)** summarizes w/o, Cand, and SEB stimulatory conditions for PBMC from *n* = 5 to 6 animals (w/o vs. Cand, *p* = 0.0271, Student’s *t*-test and w/o vs. SEB, *p* = 0.0043, Mann–Whitney test). **(C)** Analysis of CD8α coexpression of *Candida*-reactive CD154^+^CD4^+^ T cells or non-responding CD154^−^CD4^+^ T cells after Cand stimulation of PBMC. Concatenated contour plots (from *n* = 3 animals) are illustrated and numbers in gates identify CD8α negative (left rectangle gate) or positive (right rectangle gate) CD4^+^ T cells. **(D)** Flow cytometry of CD154/Cytokine coexpression analysis in either Cand (40 µg/ml) or SEB (1 µg/ml) stimulated PBMC for TNF-α, IFN-γ, and interleukin-17A. **(E)** Analysis of CD154 expression in gamma delta T cells (identified by TCR1δ^+^ expression and pregated on live CD3/duplet exclusion/FSC-SSC properties) upon antigen-specific (Cand, 40 µg/ml), superantigen-specific (SEB, 1 µg/ml), and TCR unspecific (PMA/ionomycin) stimulation.

The detection of CD154 as an early activation marker for visualizing antigen-reactive T cells by flow cytometry has been established in human and mouse studies and refined during the last years to properly identify Th cell responses to pathogens, environmental-, tumor-, neo-, or auto-antigens ranging from complex antigenic mixtures to proteins, peptide pools, or even single peptides (24, 45). To establish CD154 marker detection for antigen-reactive T cells in swine, we analyzed the Th cell response toward a complex antigenic mixture of *C. albicans* (Cand) and SEB, a bacterial superantigen known to cross-link MHC class II α-chain and TCRβ-chain. In addition, phorbol myristate acetate and ionomycin (P/I) was used as polyclonal stimulus downstream TCR triggering. Figure 1A shows CD154 expression *ex vivo* in CD4^+^ T cells from freshly isolated porcine PBMC of healthy piglets detected after 6 h of stimulation using a cross-reactive human mAb (clone 5C8). The addition of BrefeldinA during the last 4 h of stimulation prevents its transport to the cell surface and allows for intracellular staining of CD154 after fixation of cells. As shown in Figure 1A and summarized in Figure 1B, porcine CD4^+^ T cells display a defined population of CD154-expressing T cells following antigen-dependent (Cand, SEB) and TCR-independent (P/I) stimulation when compared to the unstimulated control (w/o). The frequency of *C. albicans*-specific T cells, identified as CD154^+^ cells upon antigenic stimulation, varies widely ranging from 0.07 to 0.32% (Figure 1B) of total CD4^+^ T cells from individual pigs. Similarly, the frequency of SEB-responding cells varies between individual pigs (range 0.21–1.07% of total CD4^+^, Figure 1B).

To assess whether CD154 expression assigns *C. albicans-*reactive T cells to the naïve or activated/memory T cell compartment, we analyzed CD8α coexpression on CD154^+^ and CD154^−^ cells after stimulation with *C. albicans* (Figure 1C). Our data indicates that *Candida*-specific CD154^+^ cells are enriched in the double-positive CD4^+^CD8α^+^ compartment, a subpopulation that in the pig is known to include activated and memory T helper cells (8).

A reliable marker for antigen-reactive T cells should be associated with a functional cytokine response. We therefore costained for TNF-α and IFN-γ production, known to be produced by human antigen-specific T cells in response to *C. albican*s (46, 47). Figure 1D clearly illustrates CD154 and cytokine coexpression upon SEB stimulation with most of the cytokine-producing cells expressing CD154, independent of the type of cytokine.

Furthermore, gamma delta T cells, identified as CD3^+^TCR1δ^+^ (48), do not similarly respond with CD154 upregulation to TCR-dependent (SEB or *C. albicans* lysate) or TCR-independent (P/I) stimulation when compared to CD3^+^CD4^+^ T helper cells (Figure 1E). We therefore conclude that in swine, TCR triggering induces expression of CD154 in CD4^+^ T helper cells detectable after 6 h of stimulation. Moreover, most cytokine-expressing cells coexpressed CD154 following SEB stimulation indicating that functional T cells are reliably identified by CD154 expression. Thus, we could establish CD154 as a good and promising marker for cytokine-producing antigen-reactive T cells in the pig.

### Expansion and Autologous Restimulation of Porcine Antigen-Reactive CD4^+^ T Cells

**Figure 2:**
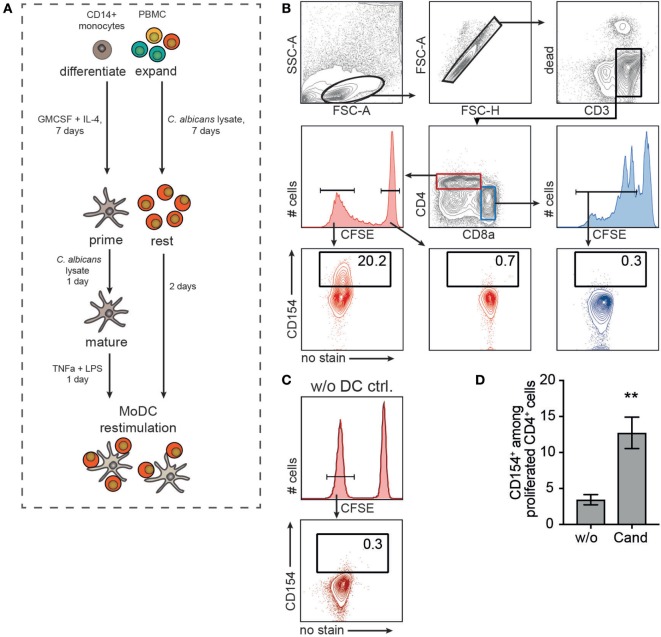
**Pig antigen-responding T cells can be expanded and visualized after monocyte-derived dendritic cell (MoDC) restimulation**. **(A)** Experimental setup. Purified blood CD14^+^ monocytes were differentiated into MoDC for 7 days in the presence of GM-CSF and interleukin-4. MoDCs were primed with 40 µg/ml *Candida albicans* lysate and maturation was triggered by TNF-α and LPS treatment for 1 day. In parallel, PBMCs were CFSE-labeled and expanded in the presence of 20 µg/ml *Candida* antigen for 7 days followed by a 2-day resting phase. Expanded lymphocytes were restimulated with autologous, primed MoDC (MoDC:T cell ratio, 1:5) and CD154 expression was assessed according to the gating strategy in **(B)**. CD154 expression of CD4^+^CFSE^low^ (proliferated cells, lower left plot) was compared to unproliferated CD4^+^CFSE^high^ (lower middle, red plot) and proliferated CD4^−^CD8α^+^CFSE^low^ T cells (lower right, blue plot). **(C)** As control, CD154^+^ frequency of Cand expanded PBMC without DC restimulation (w/o DC ctrl.) is shown exemplarily [gated on CD4^+^CFSE^low^ as depicted in panel **(B)**]. **(D)** CD154^+^ T cell frequencies after restimulating expanded T cells with unprimed (w/o) or *Candida* antigen-primed (Cand) MoDC (*n* = 6, Mann–Whitney test, **p = 0.0043).

The low frequency of antigen-specific T cells makes it difficult to study CD154 expression kinetics and its dependence on antigen dose in a detailed fashion. Therefore, it is necessary to expand the population of antigen-reactive T cells. Figure 2A illustrates a strategy that combines *in vitro* expansion of antigen-reactive PBMC with an autologous restimulation of expanded T cells using antigen-primed MoDC. PBMCs were CFSE labeled and expanded in the presence of 40 µg/ml of *C. albicans* lysate. In parallel, purified CD14^+^ blood monocytes were differentiated into dendritic cells (MoDC) using GM-CSF and IL-4 for 7 days. Rested T cells were finally restimulated with autologous, antigen primed, and matured MoDC (Figure 2A, lower panel). *C. albicans*-reactive, expanded T cells showed robust expression of CD154 when restimulated with antigen-primed MoDC (Figure 2B, lower left panel). Subgroup analysis further revealed that expression of CD154 was restricted to proliferated CD4^+^ cells (including double-positive CD4^+^CD8α^+^), with non-proliferative CD4^+^ T cells or proliferated CD4^−^CD8α^+^ T cells being negative for CD154 expression (Figure 2B). Figure 2C proofs CD154 expression of *C. albicans*-reactive, expanded T cells to be dependent on MoDC restimulation. In addition, coculturing *C. albicans* expanded T cells with unprimed (w/o) MoDC exhibited a rather low background expression suggesting that the observed CD154 expression of CD4^+^ T cells clearly depends on the presence of the specific antigen (Figure 2D). Combining expansion with antigen-primed MoDC restimulation and CD154 detection thereby precisely identified and enhanced the analysis of the antigen-reactive T cell population specific for a commensal pathogen, such as *C. albicans*.

### Time and Antigen Dose Regulate CD154 Expression of Porcine CD4^+^ T Cells

**Figure 3:**
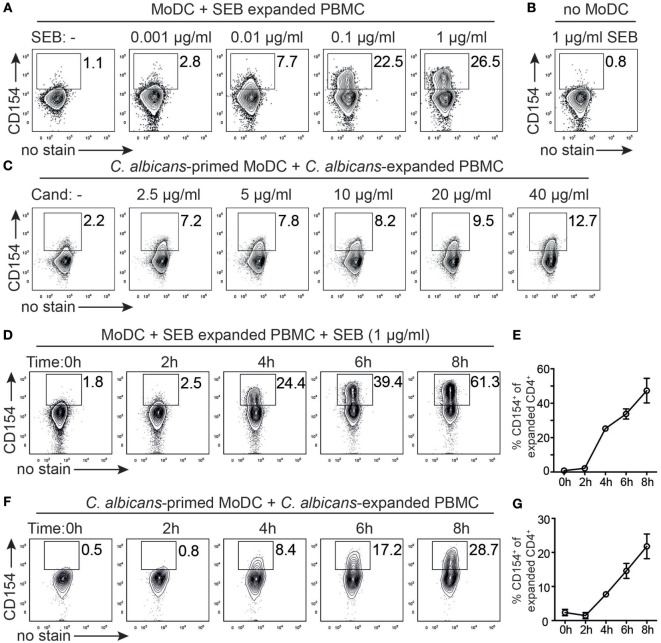
**Antigen dose and timing regulate CD154 expression of porcine CD4^+^ T cells**. **(A)** Superantigen [staphylococcal Enterotoxin B (SEB)] expanded T cells were restimulated with autologous monocyte-derived dendritic cell (MoDC) for 5 h in the absence (left plot) or presence of varying concentrations of SEB (0.001–1 µg/ml) and assessed intracellularly for CD154 expression of proliferating CD4^+^ T cells according to gating strategy of Figure 2B. **(B)** As control, SEB expanded T cells were confronted with 1 µg/ml SEB in the absence of MoDC. **(C)** *Candida albicans* antigen expanded T cells were restimulated for 6 h with MoDC that were primed with varying concentrations of *C. albicans* antigen (2.5–40 µg/ml) or left unprimed (left plot). Representative plots for two independent titration experiments are shown. **(D)** Time course of CD154 expression after restimulating SEB-expanded cells with MoDC in the presence of 1 µg/ml SEB (0–8 h). Representative plots from *n* = 3 pigs are shown and summarized in panel **(E)**. **(F)** Time course of CD154 expression following restimulation of *C. albicans*-expanded cells with *C. albicans* antigen-primed MoDC for 0–8 h. Representative plots from *n* = 3 pigs are shown and summarized in panel **(G)**. Data in panels **(E,G)** are presented as mean ± SEM of CD154^+^ T cells over time (for data points lacking error bars, SEM values are smaller than circles representing means).

For optimizing timing and dosing in the described strategy of expansion and autologous restimulation (Figures 2A,B), we first investigated whether the antigen dose had an impact on CD154 expression. Superantigen (SEB) expanded T cells where therefore restimulated for 5 h with autologous MoDC in the absence or presence of SEB (0.001–1 µg/ml, Figure 3A) and analyzed intracellular CD154 expression of proliferative CFSE^low^CD4^+^ T cells. We found an increase of CD154^+^ Th cells with increasing SEB concentrations, strongly indicating superantigen dose dependency for CD154^+^ expression. By contrast, expanded PBMC exposed to SEB (1 µg/ml) but in the absence of APCs (no MoDC, Figure 3B) did not respond with CD154 expression, consistent with the idea that CD154 expression requires TCR–MHC interaction. Similarly, a dose-dependent effect of CD154 expression was observed when restimulating *C. albicans* expanded PBMC with *C. albicans*-primed MoDC (2.5–40 µg/ml, Figure 3C). We next assessed the time course of CD154 expression in porcine T cells. Restimulating superantigen-expanded PBMC with MoDC in the presence of 1 µg/ml SEB, identified CD154^+^ antigen-responsive cells starting as early as 4 h and with increasing frequencies for all analyzed time points (0–8 h, Figures 3D,E). By contrast, before (0 h) or 2 h after restimulation, CD154 expression was negligible in proliferative CD4^+^ cells (Figure 3D and summarized in Figure 3E). A similar kinetic, but lower in ratios (Cand, 4–8 h: 7.72–21.8% mean CD154^+^ vs. SEB 4–8 h: 25.27–47.33% mean CD154^+^) was observed when analyzing *C. albicans* expanded PBMC restimulated with *C. albicans-*primed MoDC (Figures 3F,G). Notably, CD154 expression did not reach a plateau within 8 h of restimulation in both, superantigen or *C. albicans*-reactive CD4^+^ T cells. Based on these data, we defined an optimal priming dose of 40 µg/ml lysate and 6 h of restimulation time being acceptable for detecting *C. albicans*-specific T cells following MoDC restimulation. In summary, CD154 expression in swine CD4^+^ T cells was induced after short-term contact to antigen-loaded DC (4–8 h) and was clearly dependent on the antigen dose.

### Identifying Functional *C. albicans*-reactive T Cell Subsets in Healthy Pigs

**Figure 4:**
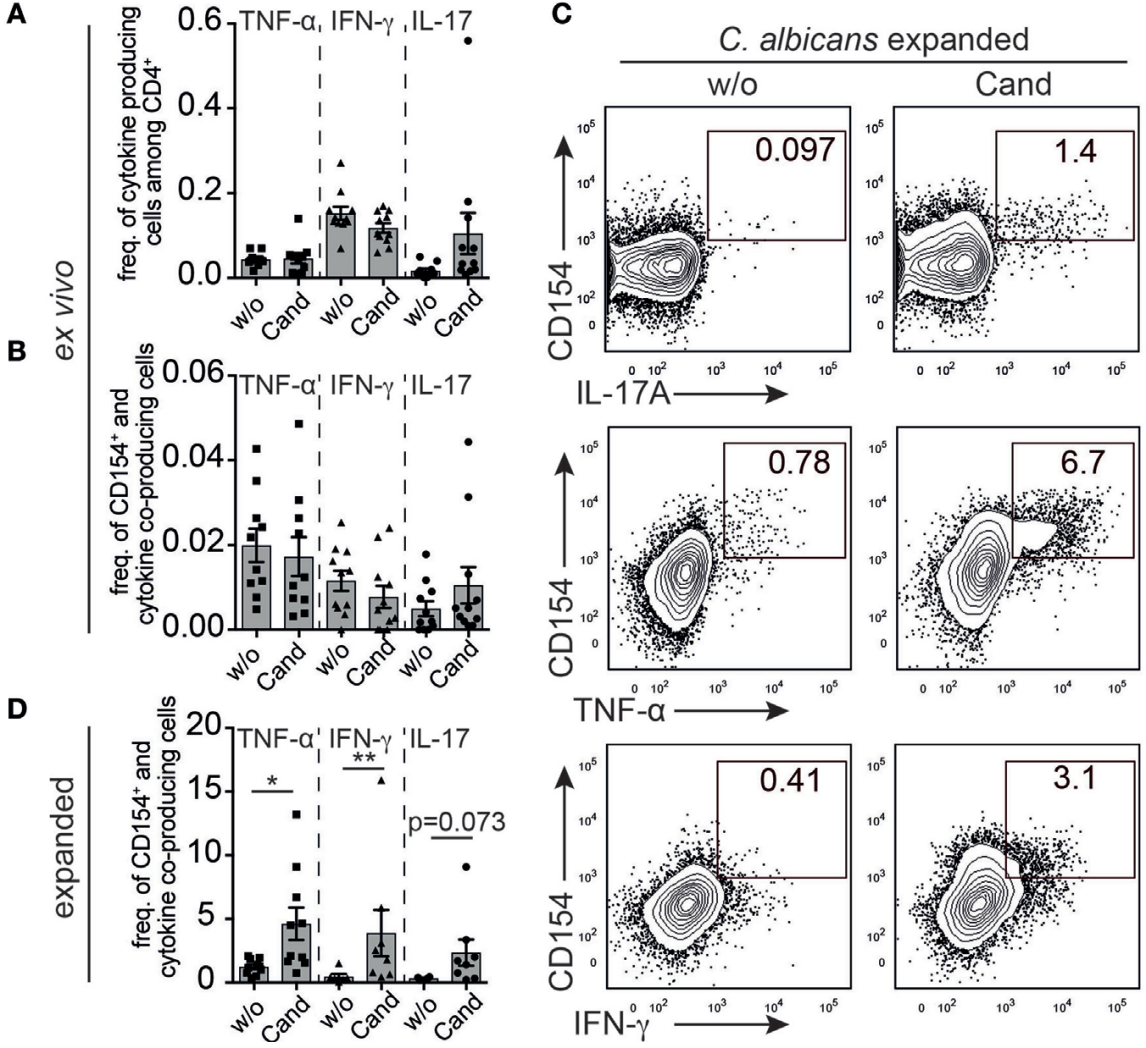
**Expansion and restimulation increases resolution in the phenotypical profile of commensal-specific T cells**. Cytokine-producing cells of porcine PBMC were directly analyzed *ex vivo* after *Candida albicans* restimulation, gated as described in Figure 1A and plotted as panel **(A)** cytokine-producing cells of total CD4^+^ T cells or panel **(B)** cytokine/CD154^+^ coproducing cells of CD4^+^ T cells (*n* = 10–11). PBMCs were further expanded for *C. albicans* antigen and analyzed for cytokine/CD154^+^ expression following restimulation with Cand-primed monocyte-derived dendritic cell (MoDC) (40 µg/ml). Panel **(C)** illustrates representative cytokine/CD154 analysis of expanded *C. albicans*-specific T cells restimulated with either umprimed (w/o) or *C. albicans*-primed MoDC. Cytokine analysis of *C. albicans*-expanded T cells are summarized in panel **(D)** (TNF-α: *n* = 10, unpaired *t*-test, *p* = 0.0166, IFN-γ: w/o *n* = 6, Cand *n* = 8, Mann–Whitney test, *p* = 0.0047, interleukin-17 w/o *n* = 4, Cand *n* = 8, Mann–Whitney test, *p* = 0.073).

T cell receptor stimulation-activated T cells start to produce a specific set of cytokines upon recognition of their cognate antigen, if they are not anergic. Quantity and quality of cytokine production greatly determines the immune response toward a specific pathogen. However, in the absence of ongoing infection, pathogen-specific cytokine producing cells from either memory or naive compartment are rare and their detection is hampered by a very low signal-to-noise ratio (22). To investigate whether the use of expansion, restimulation, and CD154 expression enhances resolution for detecting antigen-reactive cytokine producing T helper cells in healthy pigs above background signals, we first evaluated the *ex vivo* cytokine response of *C. albicans*-stimulated and non-stimulated (w/o) T cells referring to the population of either total CD4^+^ cells (Figure 4A) or depicting CD154/cytokine coexpressing CD4^+^ T cells (Figure 4B). As expected, sample heterogeneity, staining background, and limitations in processing large cell numbers restricted the analysis of rare, antigen-reactive functional subgroups and did not allow to identify precise signals above non-stimulated background (Figures 4A,B). However, porcine CD4^+^ T cells expanded in the presence of *C. albicans* lysate and cocultured with either unprimed (w/o) or antigen-primed (Cand) MoDC markedly improved resolution of antigen-responsive CD154^+^/cytokine coproducing CD4^+^ T cells (Figures 4C,D). Thus, functional subgroup analysis of antigen-reactive cells in the absence of an active infection was facilitated by increasing their numbers *in vitro* by expansion followed by autologous restimulation and analysis of cytokine/CD154 coexpression.

### Detection of Pathogen-Specific T Cells *Ex Vivo* during *A. suum* Infection

Understanding how pathogen-specific T cells orchestrate the host immune response in the early phase of an acute infection is essential for diagnostics and therapy. We therefore focused on the intestinal parasitic roundworm infection with *A. suum*, known to have a major economic impact on pig industry (49, 50). Before the parasite colonizes the intestine and completes its life cycle, it migrates along the hepato-tracheal route causing tissue damage in liver and lung. In light of its significant importance on growth performance, data on the parasite-specific adaptive immune-response in swine are sparse and urgently needed.

**Figure 5:**
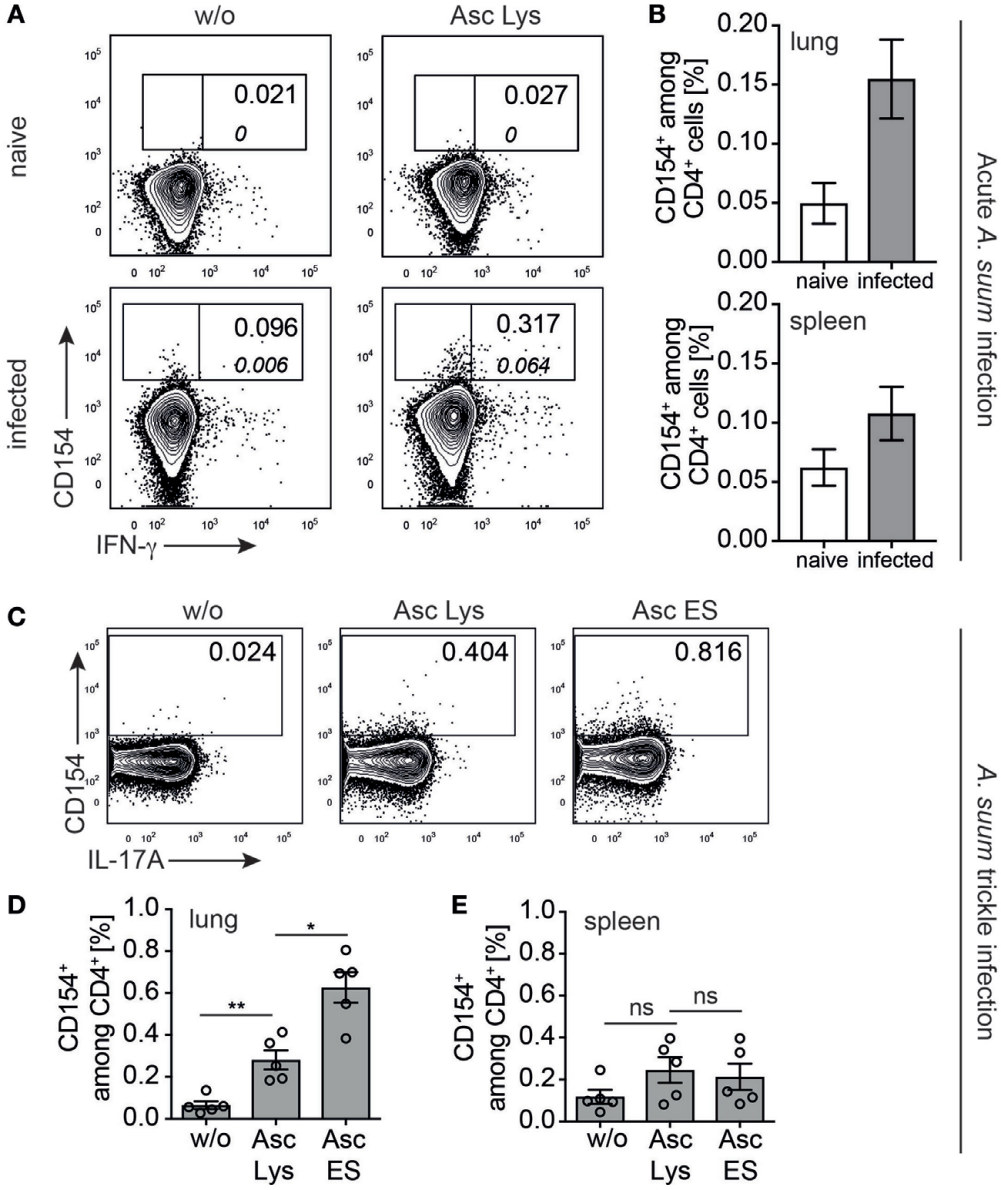
***Ascaris suum* specific CD4^+^ T cells in acute and trickle infection *ex vivo***. **(A)** Representative contour plots showing *ex vivo* analysis of CD154 expression in lung lymphocytes of naive vs. infected piglets gated on CD4^+^ T cells (according to gating strategy of Figure 1A) when left untreated or stimulated with parasite lysate of L3 larvae (Asc Lys, 20 µg/ml). *Italic numbers* indicate frequency of IFN-γ-coproducing CD154^+^CD4^+^ T cells. **(B)** Frequencies of CD154^+^ cells among CD4^+^ T cells stimulated with Asc Lys in cells isolated from lung or spleen of naive vs. infected piglets (*n* = 3, lung: median 0.0665 vs. 0.185, *p* = 0.1; spleen: median 0.0513 vs. 0.124, *p* = 0.2, Mann–Whitney test). **(C)** Representative plots from *ex vivo* lung analysis of trickle infected piglets when stimulated with worm lysate (Asc Lys, 20 µg/ml) or excretory–secretory worm products (Asc ES, 20 µg/ml) or left untreated from *n* = 5 piglets, summarized in **(D)** (w/o vs. Asc Lys: median 0.0564 vs. 0.253, *p* = 0.0079 and Asc Lys vs. Asc ES: median 0.253 vs. 0.694, *p* = 0.0159, Mann–Whitney test). **(E)** Summarized CD154^+^ frequencies of lymphocytes isolated from the spleen of trickle infected piglets, Mann–Whitney test.

We therefore asked whether CD154 expression analysis can be applied to readily identify *Ascaris*-specific T cells in affected and systemic organs of acutely infected vs. naive pigs. We analyzed *ex vivo* CD154^+^ Th cells of pigs experimentally infected with *A. suum* and necropsied at lung stage of infection (7 dpi). Lung lymphocytes were isolated and stimulated with a lysate of *A. suum* lung-stage L3 larvae (Asc Lys) for 6 h. We found an increase, yet not significant, in the frequency of antigen-induced CD154^+^ lung T cells in infected pigs that was absent in naive control animals (Figure 5A). While more *A. suum*-specific CD4^+^ T cells were detected in lungs of infected compared to naive animals (Figures 5A,B, upper panel, median 0.0665 vs. 0.185, *p* = 0.1, Mann–Whitney test), we observed only a marginal difference in lymphocytes isolated from the spleens of respective animals (Figure 5B, lower panel, median 0.0513 vs. 0.124, *p* = 0.2, Mann–Whitney test). Of note, IFN-γ-coproducing CD154^+^CD4^+^ T cells were only found in Asc Lys stimulated lung parenchymal cells of infected pigs (Figure 5A, indicated by italic numbers).

In addition to antigen-reactive T cells responding to whole worm lysate as antigen source, we aimed to determine frequencies of T cells reactive to excretory–secretory (ES) worm products, a complex mixture of released compounds with various functions, some of them known for their immunogenicity (51, 52). We therefore evaluated the frequency of *A. suum*-specific T cells in a slightly different infection scenario that more closely resembles the situation in contaminated finishing barns. Rattlerow Seghers hybrid piglets were trickle infected over 7 weeks (80 eggs/day), with incoming larvae continuously migrating to the lung for the entire infection period. To investigate against which antigenic compartment the *Ascaris*-specific response is directed, lung lymphocytes were analyzed for CD154 *ex vivo* after short-term stimulation with either *A. suum* lysate (Asc Lys) or purified and concentrated *Ascaris*-derived ES (Asc ES) products (Figure 5C). Although whole worm lysate induced a significant population of CD154^+^-expressing lung CD4^+^ T cells (w/o vs. Asc Lys median 0.0564 vs. 0.253, *p* = 0.0079, Mann–Whitney test), the frequency of *A. suum*-reactive cells stimulated with Asc ES was significantly higher (Figures 5C,D, Asc Lys vs. Asc ES median 0.253 vs. 0.694, *p* = 0.0159, Mann–Whitney test). Again, we found no substantial increase in CD154^+^-expressing splenic T cells stimulated with either Asc Lys or Asc ES (Figure 5E).

Collectively, these data demonstrate that even without prior expansion CD154-expression identified a population of *A. suum*-specific T cells in acute parasite infection (7 dpi) or following trickle infection and that worm-specific T cells preferentially accumulated in affected lung tissues.

### Detecting Vaccination Induced Antigen-Reactive Immune Responses in the Pig

To validate whether CD154 expression on swine Th cells can be used to monitor CD4^+^ T cells reactive for a single recombinant protein in a vaccination approach, we analyzed CD154 responses in immunized pigs. Here, we focused on an experimental immunization to *S. suis* regarded as one of the most important pig pathogens (35). All investigated strains of *S. suis* degrade porcine IgM (53). The responsible protease, Ide*_Ssuis_*, is a highly protective antigen against *S. suis* serotype 2 as shown in a vaccine trial with the recombinant protein (42).

**Figure 6:**
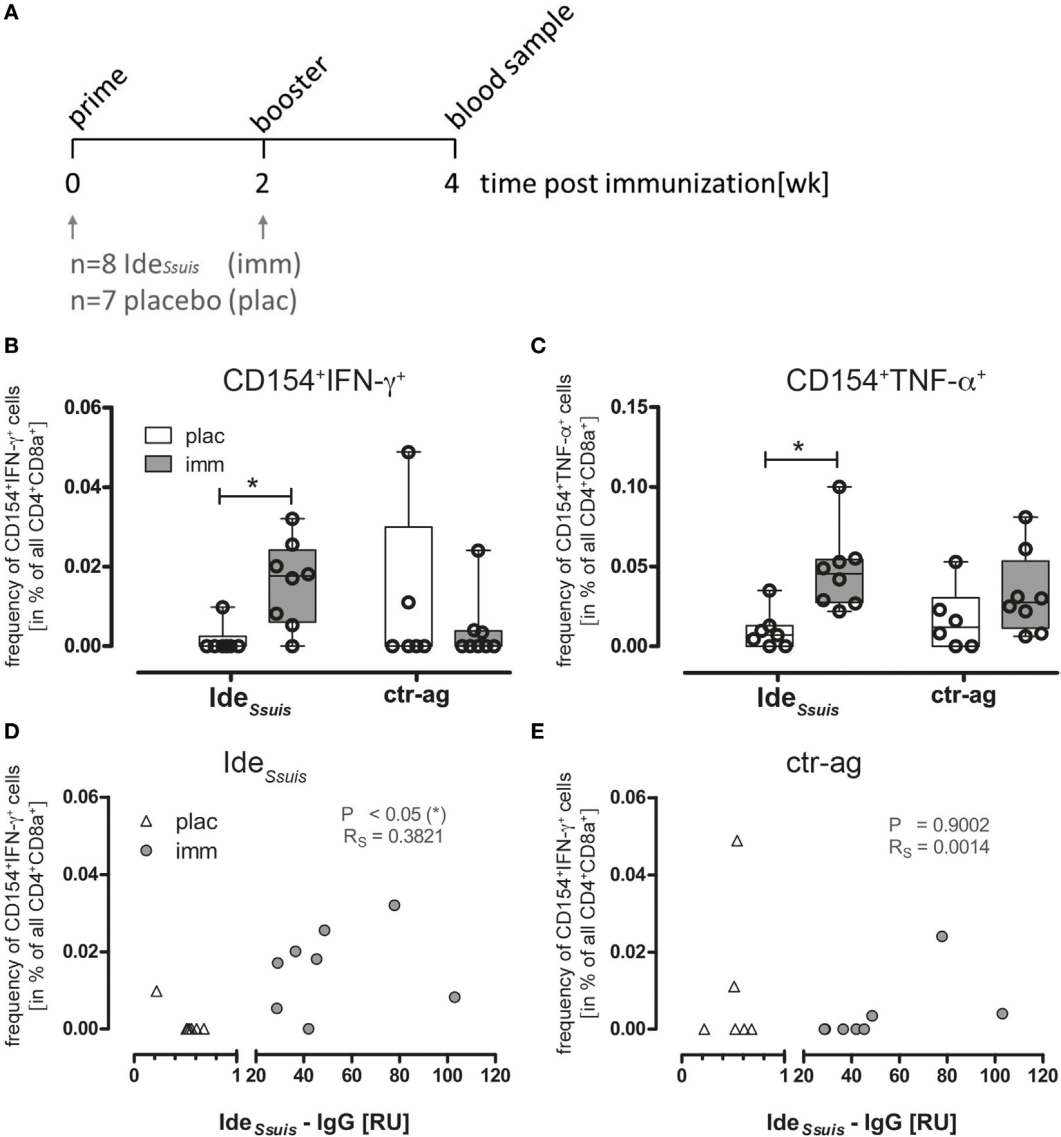
**Ide*_Ssuis_*-reactive Th cells are increased in PBMCs of immunized piglets identified by CD154 expression and cytokine production and the frequency of CD154^+^IFN-γ^+^ Th cells correlates with Ide*_Ssuis_*-specific IgG**. **(A)** Piglets were primed with rIde*_Ssuis_* followed by booster immunization 2 weeks later. Blood samples for the investigation of antigen-specific Th cells were taken 2 week post-booster immunization. **(B,C)** To detect antigen-reactive Th cells, PBMCs derived from rIde*_Ssuis_*-immunized (imm; *n* = 8) or placebo-treated (plac; *n* = 7) piglets were *ex vivo* restimulated for 18 h with 5 µg/ml rIde*_Ssuis_* or ctr-antigen (rSfb I), respectively, in presence of Brefeldin A (2 µg/ml) for the last 4 h. The frequency of antigen-specific Th cells was calculated as the difference of specified cells from antigen-restimulated and medium-cultivated PBMC, respectively. Statistical analysis was performed with Kruskal–Wallis test (**p = 0.0063) and Dunn’s multiple comparison test (*p ≤ 0.05). Ide*_Ssuis_* specific-IgGs were measured by ELISA with serum from the same piglets before and after immunization, using an independent Ide*_Ssuis_*-IgG positive reference serum. The correlation between Ide*_Ssuis_*-IgG and frequency of Ide*_Ssuis_* induced CD154^+^IFN-γ^+^ Th cells **(D)** or frequency of ctr-ag induced CD154^+^IFN-γ^+^ **(E)** was estimated by Pearson correlation (*p ≤ 0.05).

Figure 6A illustrates the experimental design for Ide*_Ssuis_* vaccination in German Landrace pigs. The animals were immunized with either recombinant Ide*_Ssuis_* or placebo (plac, adjuvant only) intramuscularly with a booster immunization 2 weeks later (Figure 6A). Two weeks after the boost, PBMCs and serum samples were obtained from both groups. Following stimulation of PBMCs (for 18 h) with medium alone, rIde*_Ssuis_* or with an unrelated recombinant protein obtained from the same expression system (ctr-ag rSfbI) pre-gated activated T helper cells (CD4^+^CD8α^+^) were further assessed for intracellular CD154 and cytokine coexpression. Ide*_Ssuis_* immunized animals showed a significant increase of IFN-γ and TNF-α secreting CD154^+^ T cells compared to placebo animals (Figures 6B,C) when stimulated with rIde*_Ssuis_*, indicating an antigen-reactive T helper cell response toward the vaccination target. Stimulation of PBMCs with ctr-ag also induced T helper cell reactivity in some of the animals but was not restricted to the immunized group. To control for polyclonal activation by residual LPS contamination during *in vitro* activation, the T cell response to 1 µg/ml LPS was analyzed in parallel and showed only background levels compared to Ide*_Ssuis_* protein (data not shown). A successful vaccination requires activation of T helper cells capable of costimulating B cells for generating antibodies that protect against the disease of interest. We analyzed Ide*_Ssuis_*-specific IgG antibody titers in immunized animals 2-week post-vaccination to correlate frequency of CD154^+^/IFN-γ producing T helper cells with the Ide*_Ssuis_*-specific IgGs, since especially IFN-γ is important to support the IgG class-switch of B cells (54). Indeed, we found a significant correlation of Ide*_Ssuis_*-specific IgG and frequencies of Ide*_Ssuis_*-specific, IFN-γ producing CD154^+^ Th cells in immunized animals (Figure 6D). By contrast, no correlation was observed when plotting Ide*_Ssuis_* IgG levels against ctr-ag-specific CD154^+^ Th cells (Sfbl, Figure 6E). These data suggest that the analysis of CD154^+^ expression can be used to identify immunization-responsive cells and can therefore be applied to validate the induction of a T helper cell response toward a given vaccination target in swine.

## Discussion

Human and mouse studies have shown that T cell receptor activated CD4^+^ cells start expressing CD154 (CD40L) early after antigen recognition, transient and independent of their phenotypical differentiation status, revealing the total pool of antigen reactive T cells directly *ex vivo* (22, 25, 27, 55). We here established CD154 detection on antigen-reactive CD4^+^ T cells in the pig as a human-relevant large animal model using a cross-reactive, anti-human CD154 antibody (clone 5C8) and intracellular staining. Our *ex vivo* analysis of pig PBMC demonstrated that CD154 is expressed in CD4^+^ T cells upon stimulation with bacterial superantigen (SEB) or lysates of a commensal fungi (*C. albicans*) but also after TCR-independent P/I stimulation. However, with the current tools available at the moment, we could not directly prove SLAII-TCR interaction as demonstrated in mouse and human studies (27, 31). But, consistent with studies on human PBMC, we found CD154 to be coexpressed with functionally important cytokines such as IFN-γ or TNF-α (46, 47). Regarding the percentage of *C. albicans* responsive T cells from the entire CD4^+^ T cell pool, our data are comparable to recent human studies reporting *C. albicans*-specific responses ranging from of 0.1 to 0.2% of total CD4^+^ (22) from in freshly isolated PBMC from healthy individuals.

A well-studied peculiarity of porcine T helper cells is the abundant expression of CD8α on a substantial proportion of CD4^+^ T cells (8, 56, 57). Ontogenetic studies, and also studies evaluating proliferative capacity, *de novo* stimulation, or antigen-induced cytokine secretion for the different CD4^+^ subpopulations concluded, that CD4^+^CD8α^+^ T cells resemble activated or memory CD4^+^ T cells (8). We therefore asked whether pig fungus-specific CD154^+^ T cells derive from the naïve (CD4^+^CD8α^−^) or effector/memory compartment (CD4^+^CD8α^+^) and found the majority of reacting cells to cluster in the latter one, indicating that those are predominantly antigen-experienced cells, but that also naïve, antigen-reactive cells, potentially being activated for the first time *in vitro*, can be detected using CD154^+^ expression. Whether pig CD154^+^ Th cells display different memory phenotypes deserves further investigation by detailed subgroup analysis of CD154^+^ combined with CD8α, CD45RC, SLAII, and CD27 as demonstrated by other groups (14). As the second special feature and again in contrast to human and mouse species, γδ T cells are not a minor but a rather abundant population of blood circulating lymphocytes in swine (48, 58) and their role, functions, and target cells are still not completely clarified. It was therefore important to prove that CD154 expression is restricted to the αβ T cell compartment, even when circumventing to directly trigger the T cell receptor.

In the second step, we amplified antigen-responsive cells by *in vitro* expansion to optimize and study time- and dose-dependent responses in more detail during autologous, antigen-specific restimulation. CFSE-dilution analysis of the expansion culture allowed for a subgroup analysis to characterize cells that subsequently expressed CD154 upon restimulation. Our data on fungal-specific expansion clearly demonstrated restriction of the CD154 signal to proliferated CD4^+^ T cells, but not to non-proliferated or proliferated CD4^−^CD8α^+^ T cells, again emphasizing the relevance of CD154 as an T helper cell specific activation marker.

Using expansion and restimulation, our results indicate that both superantigen-activated and fungus-induced CD154 expression follow a similar kinetic, starting between 2 and 4 h after antigenic stimulation. This is in line with kinetic studies with mouse (30, 55) and human PBMCs (26). Notably, in contrast to those studies we found no indication that CD154 expression peaked around 6 h after restimulation in expanded swine CD4 T cells. But, 6 h after MoDC restimulation, we already found a substantial population of antigen-responsive cells that was sufficient for downstream phenotyping of fungus-reactive T cells and therefore avoided longer restimulation times. It should be noted that restimulation times and antigen dosing have to be carefully adapted according to any pathogen, infection stage, or sample handling (e.g., working with fresh or frozen material). For priming MoDC with *C. albicans* before restimulation, we determined an optimal priming dose of 40 µg/ml lysate with higher dosing not substantially increasing the frequency of CD154-expressing cells (data not shown).

For a basic understanding and also for modulation or intervention of the pathogen-specific T cell response, characterizing the responding T cell repertoire is crucial. The detection of cytokines enables identification of functional subsets of reactive cells. In a recent swine study, Talker and colleagues thoroughly assessed multifunctional, virus-specific CD4^+^ and CD8α^+^ T cells in influenza virus infected pigs *ex vivo*. Their data highlight the rareness of antigen-activated cytokine producers detectable in peripheral blood, never exceeding 0.5% cytokine producer of total CD4^+^ T cells throughout the course of an acute, experimental virus infection (9, 34). However, defining antigen specificity based on preselected cytokines may potentially underestimate the complete frequency and complexity of antigen-specific T cell responses.

Similarly, in the absence of an acute infection data from human PBMC demonstrated percentages of pathogen-specific CD154^+^CD4^+^ T cells ranging from 0.03 to 0.64% and irrespective of the cytokines they are producing, referring to defined antigens of the commensals *A. fumigatus, C. albicans*, or common viruses such as cytomegalovirus or adenovirus (22). Our data on the T cell response of outbred pigs to *C. albicans* revealed a high signal-to-noise ratio that hampered a comprehensive *ex vivo* cytokine profiling. However, when we amplified antigen-responsive T cells by expansion and analyzed the T cell response during antigen-primed MoDC restimulation, we were able to increase the resolution between background levels and antigen-induced cytokine producers. While our assay offers advantages to assess CD4^+^ T cells specific for defined antigens and in sufficient numbers for further phenotypic analysis and even more, allows to predict unresponsive, e.g., anergic T cells that would escape cytokine-based detection (23), it might be argued that phenotype and function can be altered after expansion by means of outgrowth (e.g., selective expansion of specific clones) or *in vitro* modification of effector functions (59).

One very important benefit of using a human-relevant large animal model is the possibility to easily dissect organ tissue to study immune cells that are directly in contact with a given pathogen. We therefore selected a parasitic infection that not only has a significant, economic impact in pig production but also is zoonotic and highly prevalent in human populations in tropical and subtropical areas with poor hygiene management—the roundworm *Ascaris* sp. (36, 49, 50). A major hallmark of *Ascaris* sp. infection is the tissue migratory phase, when larvae that penetrated the cecal wall travel *via* the liver and the blood stream into the lung and from there, by being coughed up and swallowed down, back into the small intestine. A massive larval infiltration of the lung causes tissue destruction, pulmonary eosinophilia, and infiltration of inflammatory cells that can lead to acute pneumonia (50). By analyzing the pathogen-specific T cell responses during experimental *A. suum* infections, our data reveal three important points: First, from a technical perspective, we could prove that even after long-lasting enzymatic digestion to isolate pig lung lymphocytes, CD154 expression can be used to study TCR-mediated responses under physiological conditions of antigenic stimulation. Second, during larval migration in acute and also in trickle infection, worm-specific CD4^+^ T cells accumulated in the organ being naturally affected. And third, comparing whole worm lysates with worm-derived excretory–secretory products as antigenic source, the latter exhibited a higher frequency of antigen-responding cells identified in affected lungs after trickle infection. These findings could be explained by recalling that proteins released by live parasites are directly targeting host barrier and host immune cells contain highly immunogenic antigens and are known to potently interfere with every aspect of host immunity (60). Of note, the development of pig-specific antibodies to target Th2-related cytokines (IL-4, IL-13, or IL-5) would greatly enhance the characterization of worm-specific CD4^+^ T cell responses.

For the first time, we could identify antigen-reactive T helper cells by immunization with a recombinant antigen of *S. suis*. In previous *S. suis* studies, the serological immune response in pig against recombinant antigens was well characterized (42, 61, 62). But until now, data about T cell response in *S. suis* infected pigs are lacking. A recent study investigated the CD4^+^ T cell response in systemically *S. suis*-infected mice and demonstrated CD4 KO mice to display a reduced survival (63). To control for Ide_Ssuis_-specific T helper cell detection by CD154 expression, we used an available His-tagged control antigen (ctr-ag), namely SfbI of *S. pyogenes*, that is not known to have a homolog in a porcine pathogen including *S. suis*. Though not significant, we observed a putative T cell reactivity to this protein. Noteworthy, conserved T-cell epitopes especially in the C-terminal fibronectin-binding domain of SfbI were identified, recognized by three different mouse strains (64). Therefore, we suggest a possible cross-reactivity with other fibronectin-binding proteins (65) expressed by bacteria on the mucosal surfaces of those piglets that show reactivity toward SfbI. Thus, application of CD154 expression following antigen-specific stimulation as a marker for protein-specific T helper cells will provide new perspectives to investigate vaccination-responsive T cell immunity in pig.

Collectively, our data offer new ways to assess and characterize rare antigen-reactive CD4^+^ T cell in pigs, a vital component in understanding disease pathogenesis and immunity in pigs as natural hosts and as large animal models for human infectious disease that was currently lacking in swine immunology research.

## Ethics Statement

All animal studies were performed according to the principles outlined in the European Convention for the Protection of Vertebrate Animals used for Experimental and other Scientific Purposes. Ethical approval was obtained from the Landesamt für Gesundheit und Soziales Berlin, Germany (LaGeSo) for sampling blood from healthy pigs (regulation number G0037/16, L0363/08, A0100/16) and for acute parasite infection (H0288/15), from the Ethical Committee of the Faculty of Veterinary Medicine, Ghent University, Belgium for the parasite trickle infection study (EC2015/55), and from the Landesdirektion Sachsen, Leipzig, Germany for Immunoglobulin M-degrading enzyme of *S. suis* (Ide*Ssuis*) immunization studies (regulation number TVV14/15, N01/16).

## Author Contributions

FE designed experiments, carried out experiments, evaluated data, and wrote the manuscript. PS, SS, NS, PG, CB, and RP performed experiments. SH, GA, JZ, PG, and CB designed parts of the animal studies. SH, PS, SS, GA, and CB wrote the manuscript.

## Conflict of Interest Statement

The authors declare that the research was conducted in the absence of any commercial or financial relationships that could be construed as a potential conflict of interest.
